# Deep Learning to Detect Pancreatic Cystic Lesions on Abdominal Computed Tomography Scans: Development and Validation Study

**DOI:** 10.2196/40702

**Published:** 2023-03-17

**Authors:** Maria Montserrat Duh, Neus Torra-Ferrer, Meritxell Riera-Marín, Dídac Cumelles, Júlia Rodríguez-Comas, Javier García López, Mª Teresa Fernández Planas

**Affiliations:** 1 Department of Radiology Consorci Sanitari del Maresme (Hospital de Mataró) Mataró Spain; 2 Scientific and Technical Department Sycai Technologies SL Barcelona Spain

**Keywords:** deep learning, pancreatic cystic lesion, neural networks, precursor lesions, pancreatic cancer, computed tomography, magnetic resonance, cancer, radiologist, technology

## Abstract

**Background:**

Pancreatic cystic lesions (PCLs) are frequent and underreported incidental findings on computed tomography (CT) scans and can evolve to pancreatic cancer—the most lethal cancer, with less than 5 months of life expectancy.

**Objective:**

The aim of this study was to develop and validate an artificial deep neural network (attention gate U-Net, also named “AGNet”) for automated detection of PCLs. This kind of technology can help radiologists to cope with an increasing demand of cross-sectional imaging tests and increase the number of PCLs incidentally detected, thus increasing the early detection of pancreatic cancer.

**Methods:**

We adapted and evaluated an algorithm based on an attention gate U-Net architecture for automated detection of PCL on CTs. A total of 335 abdominal CTs with PCLs and control cases were manually segmented in 3D by 2 radiologists with over 10 years of experience in consensus with a board-certified radiologist specialized in abdominal radiology. This information was used to train a neural network for segmentation followed by a postprocessing pipeline that filtered the results of the network and applied some physical constraints, such as the expected position of the pancreas, to minimize the number of false positives.

**Results:**

Of 335 studies included in this study, 297 had a PCL, including serous cystadenoma, intraductal pseudopapillary mucinous neoplasia, mucinous cystic neoplasm, and pseudocysts . The Shannon Index of the chosen data set was 0.991 with an evenness of 0.902. The mean sensitivity obtained in the detection of these lesions was 93.1% (SD 0.1%), and the specificity was 81.8% (SD 0.1%).

**Conclusions:**

This study shows a good performance of an automated artificial deep neural network in the detection of PCL on both noncontrast- and contrast-enhanced abdominal CT scans.

## Introduction

Pancreatic cancer is one of the most frequent and aggressive cancers in the digestive tract, being the fourth leading cause of death by cancer in Europe [1,2]. Due to its lack of specific symptoms and signs, most patients are detected in an advanced stage. The current average 5-year survival rate is 9%, and it depends critically on when the cancer is detected. Indeed, this 5-year survival rate varies by more than 30% when the cancer is detected in a phase where it can still be surgically removed and when the cancer has already spread to other tissues in the body [3].

This type of cancer can originate from precursor cystic lesions [4]. Pancreatic cystic lesions (PCL) are increasingly common incidental findings on abdominal imaging tests. Studies have shown that up to 70% of PCLs are diagnosed incidentally on computed tomography (CT) scans due to unrelated symptoms, making CT scans the first accessible source of information. These previously undetected cystic lesions are found on 3% of abdominal CT examinations [5,6] and 13%-21% of abdominal magnetic resonance imaging studies [7,8]. However, autopsy studies have evidenced a much higher prevalence, revealing that up to 50% of the older population may present at least one pancreatic cyst [6].

PCLs have a wide diversity, and their differential diagnosis includes nonneoplastic cysts (pseudocysts) and neoplastic ones. Neoplastic lesions encompass benign lesions, such as serous cystadenomas (SCA), to mucinous lesions, such as mucinous cystic neoplasms (MCN), and intraductal papillary mucinous neoplasm (IPMN), which may progress to PC. Therefore, identifying precancerous mucin–producing cysts offers a unique opportunity for early detection and prevention of PC. Once a PCL is found, patients are recommended to follow up a lifelong surveillance program with imaging modalities (magnetic resonance imaging or CT) to identify early-stage cancer or high-grade dysplasia [9,10]. Consequently, correct management of PCL may prevent progression to pancreatic cancer, while reducing the need for lifelong screening and related costs.

In this complex scenario, automated detection of pancreatic precursor lesions could increase the detection of this underreported entity and help with a proper surveillance of these patients. A limited number of publications regarding this topic have been released in recent years, most of them in an experimental offline setting and applying different methodologies [11]. Additionally, although existing methods of automated analysis have shown to be accurate for images of individual organs, they still struggle to deal with the variability of structures, shape, and location of abdominal organs [12]. Artificial intelligence (AI)–based algorithms have shown promising results in the detection of preneoplastic lesions in the pancreas [13,14], but they are still far from implementation in the clinical practice.

The aim of this study was to develop and test an artificial deep neural network (AGNet) [15] for automated detection of PCLs. This kind of technology can help radiologists to cope with an increasing demand of cross-sectional imaging tests and increase the number of PCLs incidentally detected, thus increasing the early detection of pancreatic cancer.

## Methods

### Ethical Considerations

Our research adhered to the ethical principles outlined in the 1975 Declaration of Helsinki. The data used in this study were retrospective and anonymized. The study was approved by the hospital Institutional Ethical Review Board under code 90/20 as an observational retrospective single-center study, and the requirement for informed consent was waived.

### Study Population

A total of 297 abdominal, thoracoabdominal, or pelvic CT scans acquired at Hospital de Mataró between 2010 and 2021 and diagnosed with a PCL as well as 38 CT scans as controls were selected for the study. All CT scan images were subjectively checked for quality and absence of relevant respiratory artifacts, which could cause misdiagnosis in the abdominal region. The exclusion criteria were underaged patients, artifacts or bad quality in the CT scan image, and patients having undergone surgery in the past to treat the PCL and having a prothesis in the pancreas that affects the image. Importantly, patients diagnosed with pancreatic adenocarcinoma or any kind of tumor in the pancreas were also excluded from the study.

Of note, a CT image is considered “bad quality” if there is movement or blurriness in it (mostly in the abdominal area, where the pancreas is located). Studies that included these types of images were excluded from the training and testing set because they would impact the learning process of the network or the testing in a negative way, which could then lead to false negatives or false positives.

The final study population consisted of 136 patients: 73 male (178 studies; mean age 67.75, SD 10.74 years) and 63 female (157 studies; mean age 73.52, SD 10.67 years). A mean of 2 (SD 1.4) CT studies and a median of 2.4 studies were available per patient.

### Patients’ Characteristics

From the whole cohort of 136 patients, 9 (6.5%) of them had a confirmed diagnosis through endoscopic ultrasound–guided fine needle aspiration or surgical resection of the lesion. In the other 16 patients, no material or insufficient yield was extracted to evaluate the specimen. The rest of the patients were diagnosed by a minimum of 2 experienced radiologists, taking into consideration the complete clinical record and the evolution of the patient.

**Figure 1:**
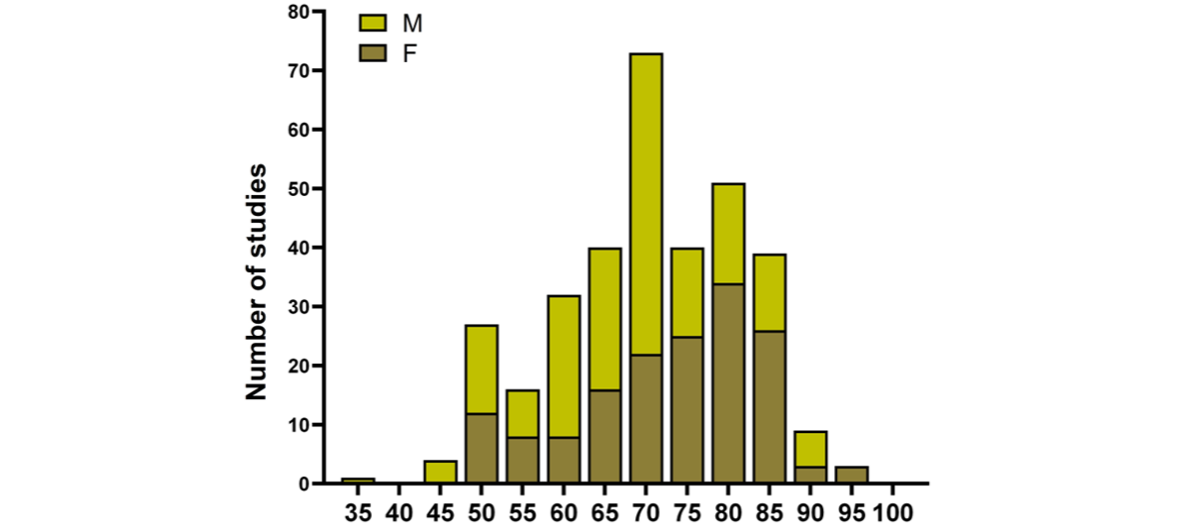
Number of studies (CT scans) with pancreatic cystic lesion distributed by age and sex (x-axis).

Patients with the following PCLs were included in the study: IPMN, MCN, SCA, and pseudocysts. A total of 14 (4.2%) of the lesions were not classified in the above classification due to unspecified imaging characteristics and were categorized as cyst (Table 1). The number of studies (CT scans) with PCLs distributed by age and sex is shown in Figure 1.

Data sets were further divided between the training set (a subset to train the model) and the testing set (a subset to test the trained model). The final training data set comprised 93 patients, representing a total of 241 CT scans, and the final testing data set comprised 43 patients, representing a total of 94 CT scans. PCLs were distributed proportionally in both data sets.

**Table 1:** Diagnostic distribution per the study.

Diagnosis	Values, n (%)
Serous cystadenoma	42 (12.5)
Intraductal papillary mucinous neoplasm	154 (46)
Mucinous cystic neoplasms	5 (1.5)
Pseudocyst	82 (24.5)
Cyst	14 (4.2)
No cyst	38 (11.3)

#### CT Protocols

CT examinations were performed with a GE BrightSpeed 16 slice CT scanner (GE Healthcare). Slice thickness was between 1.25 mm and 5 mm. Mean tube current was 440 mA, and the mean peak kilovoltage was 340 (SD 40) kVp. Contrast agent was administered with injection rates ranging from 2.5 to 3 mL/s, using Omnipaque or iomeron (both 300 mg iodine per mL).

The protocols included in this research had the following characteristics:

- From lung bases to pubic symphysis, 2 helixes are made at 30 and 65 seconds after the injection of 100 mL of the solution (30 mL of iodine), preceded and followed by 20 mL of physiological solution.
- Two helixes are made from the base of the neck to the lower edge of the liver and from the pulmonary bases to the pubic symphysis after the injection of the exposure value contrast. In this case, 120 mL of solution is injected.
- From lung bases to pubic symphysis, 1 helix is made at 65 seconds after the injection of 100 mL of the solution (30 mL of iodine), preceded and followed by 20 mL of physiological solution.

#### Image Analysis

CT scan images were exported anonymously in Digital Imaging and Communication on Medicine format from the picture archiving and communication system of the hospital. Digital Imaging and Communication on Medicine files were converted to Neuroimaging Informatics Technology Initiative files (using dicom2nii software; version from August 4, 2014; University of South Carolina). Two radiologists (NTF and MMD) with 11 and 20 years of experience manually drew, slice by slice, the region of interest, delimiting the pancreatic cysts found in the image using the open-source software 3D Slicer (version 4.11) [16]. Each radiologist segmented all cases used in the study and checked the segmentation performed by the other radiologist. Any discrepancies between the authors were resolved through discussion with the presence of a third reviewer (MTFP), until consensus was reached.

The preprocessing steps included the application of filters and registration to improve and harmonize image quality across CT scans.

First, a soft-tissue normalization [17] was applied. After studying the pixel distribution of 100 CTs of the data set, it was observed and confirmed by the state of the art that the Hounsfield unit (HU) of the pancreas is centered around 50, and most of the cystic lesions were close to this value as well. Hence, to eliminate the irrelevant parts of the abdomen and highlight the main features for the study, the soft-tissue normalization was centered in 50 HU, and a windowing length of ±100 around 50 HU was applied.

**Figure 2:**
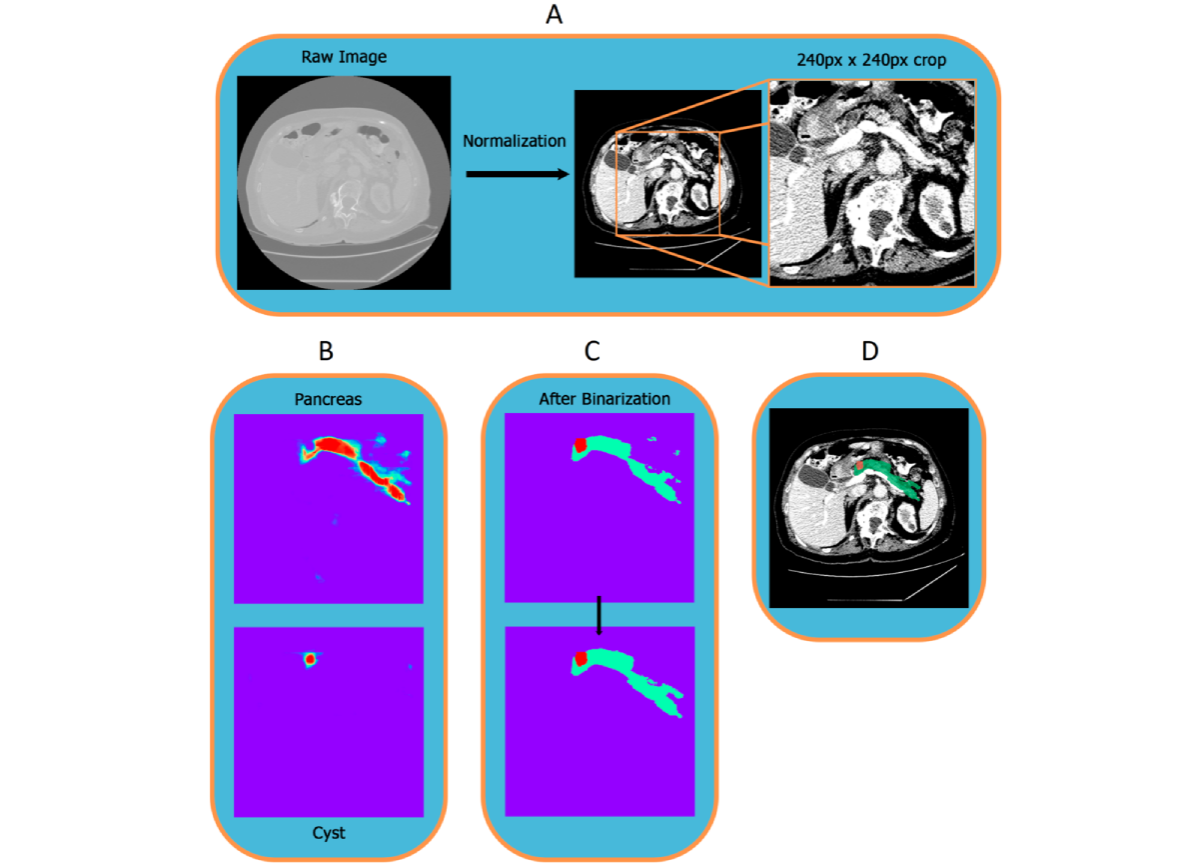
Diagram of the steps implemented in the pipeline. (A) Preprocessing. (B) Logits. (C) Postprocessing. (D) Output.

Afterwards, a central cropping of the CTs was performed, only keeping the center of the abdomen, where the pancreas is supposed to be. The cropping was not too harsh to avoid the possibility of eliminating the pancreas from the CT image being used for the following semantic segmentation study. The image analysis pipeline is depicted in Figure 2.

#### Model Training

**Figure 3:**
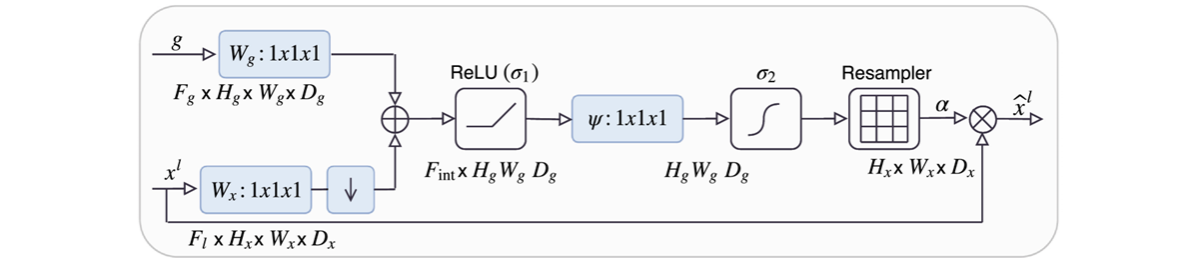
Illustration of the additive attention gate [15]. Reproduced from the cited source which is published under Creative Commons Attribution 4.0 International License [21].

The neural network used for this study was the AGNet [15]. The main structure was a basic UNet [18] with skip connections and additive attention gates (AGs). The input image was downsampled, using max-pooling, by factor 2 at each scale in the encoding part and trilinearly upsampled by the same factor in the decoding part. In each stage of the encoding-decoding architecture, a skip connection from the corresponding encoding stage to the corresponding decoding stage was added. This skip connection enters to the AG together with the output of the previous decoding stage. Thanks to this skip connections using coarser information, we are able to model the location and the relationship between tissues at a global scale. The architecture of the AG is shown in Figure 3.

**Figure 4:**
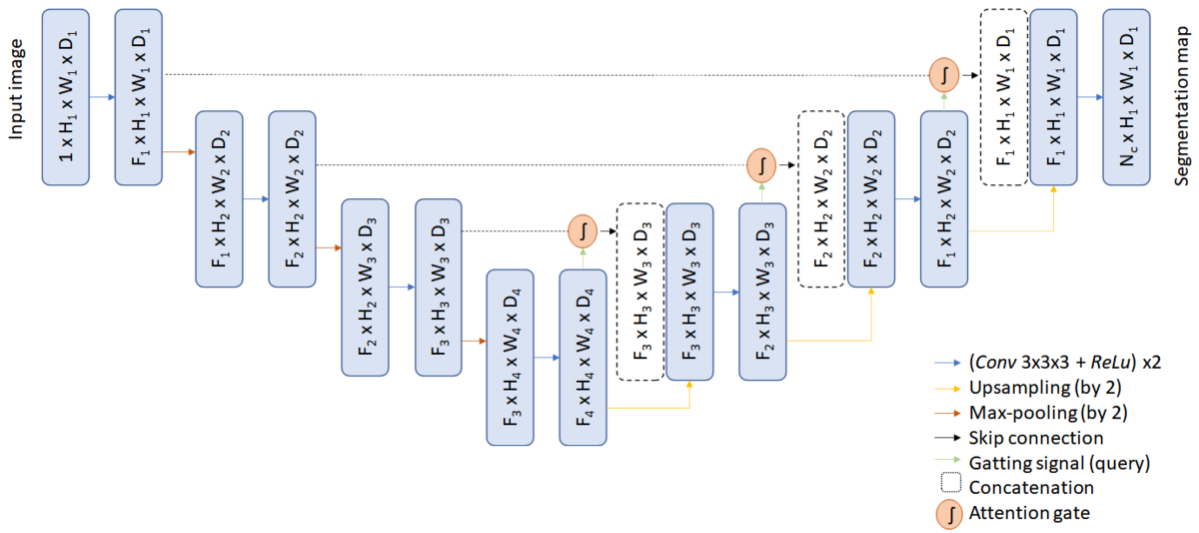
Scheme of the deep neural network architecture [15]. Fl: Feature map in the layer l; H: height; W: width; D: dimension; Conv 3x3: convolution operation with a 3x3 kernel; ReLu: rectified linear unit operation. Reproduced from the cited source which is published under Creative Commons Attribution 4.0 International License [21].

The output of these AGs was the element-wise multiplication of the attention coefficients (*α*) and the intput feature maps came from the previous stage of the decoding part (*x*; Figure 4). Attention coefficients were used to identify salient regions and preserve only activations that are relevant. There is one attention coefficient computed for each pixel vector 
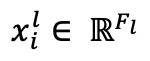
, where *F^l^* corresponds to the feature maps in layer . In the case of this study, there are multidimensional attention coefficients, each dimension corresponding to one class. The other input of the AG was a gating vector 
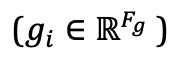
, which contained contextual information to determine focus regions. The AGs used were additive since addition between the gating signal and the feature maps were used to obtain the attention coefficients.

The network was trained for 700 epochs and had a batch size of 4. The training was performed with over 430 3D CT studies. The algorithm of optimization used was Adam [19]. The Adam algorithm is an adaptive gradient algorithm that adapts the value of the learning rate if the network does not improve the performance during training. We set the threshold of learning rate modification after 30 epochs, and it decayed 1e-6. The initial learning rate was set to 1e-4.

The initialization weights’ algorithm used was Kaiming [19,20], and the loss function used was the dice coefficient for multiclass segmentation.

## Results

The goal of this work was to implement a pipeline for PCLs detection on CT scan images as well as the pancreas. This was performed with a two-step pipeline formed by a first preprocessing that consisted of a normalization of all the data sets with a soft tissue normalization technique centered at an HU of 50. This value was selected since it is the state-of-the-art value assigned to the organs and it matches with the mean HU of the pancreas calculated for all the studies in our data set. Afterwards a central crop of the CT was applied; from a slice size of 512×512 to 240×240 after the central cropping to just focus on the center of the abdomen (anatomic location of the pancreas). Finally, the network was trained with random patches of 160×160 of this central crop, and therefore, the inference consisted of iterating around this central crop of multiple inferences of patches of 160×160.

During the inference, the test-time augmentation (TTA) technique was applied. For every CT, 4 geometrical transformations were used. Multiple options were considered in which way the TTA should be applied; however, we concluded that translation and rotation transformations were the most accurate since, for example, flipping would just confuse the network. Hence, after studying multiple options, a positive rotation of 7 degrees and a negative rotation of 11 degrees as well as 2 positive translations of 5 and 10 pixels were considered. Positive and negative rotations were considered since in CT scans the abdomen can be tilted one way and the other, but higher values for both rotation and translation would just result in bad predictions. Using more TTA transformations were ruled out due to the latency that this adds to the final pipeline. The final result is a merging of this 4 TTA transformations inferred and the original CT without any transformation. We averaged the probability of each class, and after having them merged, a softmax function was applied for obtaining the final binarized image [22].

**Figure 5:**
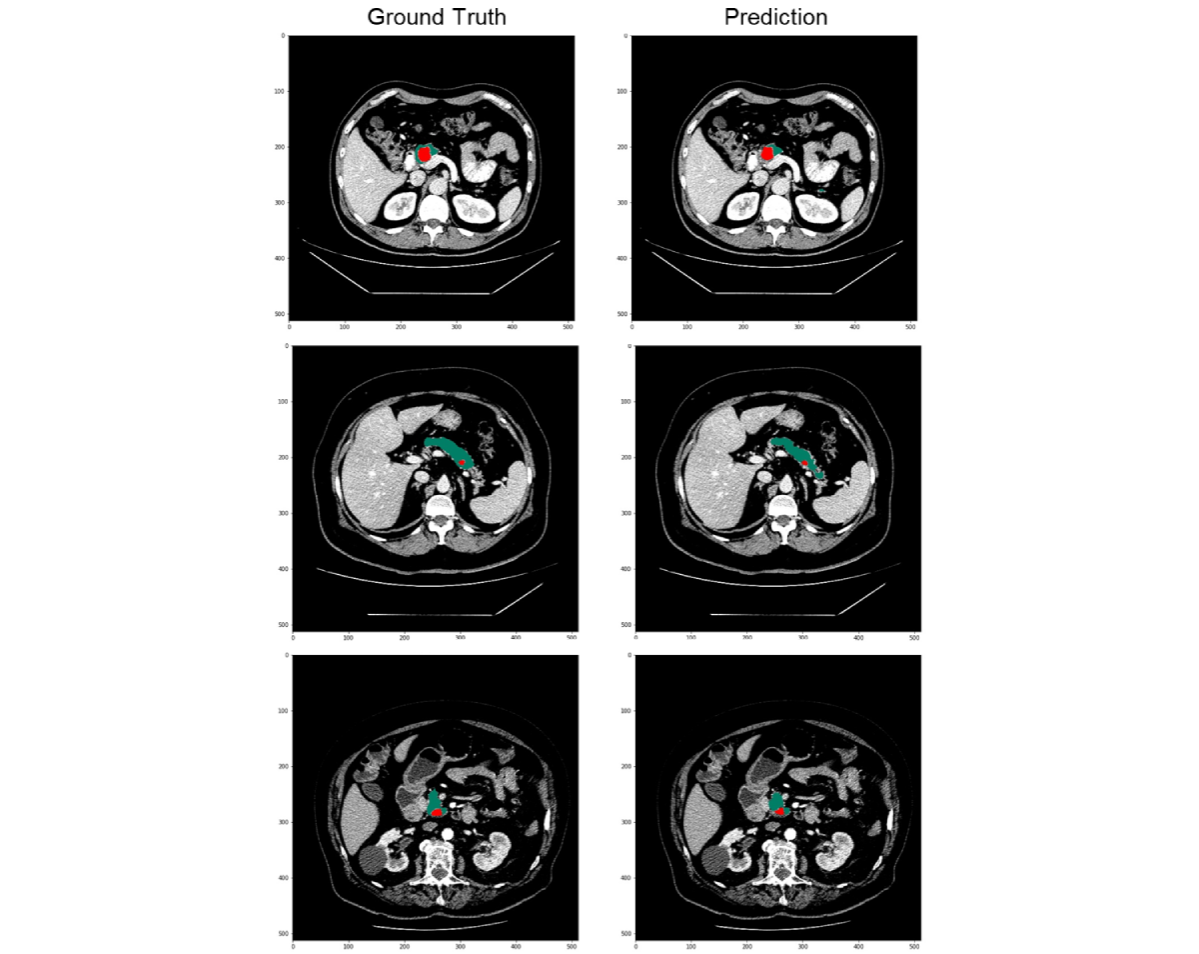
Illustration of the qualitative results obtained. Each pair of images belongs to a patient with a pancreatic cystic lesion. The left image of the pair is the ground truth, while the right one is the outcome of this method. The pixels that belong to the pancreas are painted in green and the ones for the pancreatic cystic lesion in red.

Finally, a postprocessing pipeline was implemented to improve the segmentation results performed by the network and minimize the number of false positive detections. First, a mask of the abdomen was generated and eroded to eliminate wrong predictions in the edges of the abdomen, where the pancreas anatomically is not found. Secondly, all segmented cysts that were not in touch with the pancreas were also removed. Finally, we established a minimum of 10 voxels to consider a predicted cyst as true positive. Therefore, if there were some randomly segmented pixels considered as cysts that were not previously filtered, they were ignored. Images with qualitative results of this method are shown in Figure 5.

The fully automated segmentation was performed on a modern computer with an NVIDIA GPU T4 to automatically detect PCLs in abdominal CT scans. The programming language used was Python and the framework for the model development was PyTorch. The sensitivity for all cases was 93.1% (SD 0.1%), and the specificity was 81.8% (SD 0.1%).

**Figure 6:**
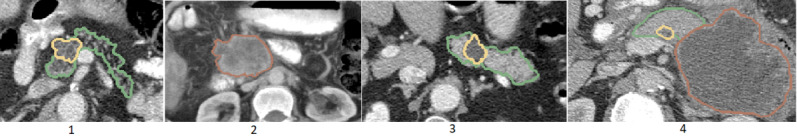
Example of the types of pancreatic cysts included in this research. (1) Serous cystadenoma, marked in yellow. (2) Mucinous cystic neoplasm, marked in red. (3) Intraductal papillary mucinous neoplasm, marked in yellow. (4) Pseudocyst, marked in red). Pancreas is depicted in green.

Additionally, due to the small amount of some subtypes of pancreatic cysts in the training database (Figure 6), we considered it reasonable to divide the whole cohort of patients into 2 big groups: on the one hand, the most dangerous cyst types, bearing malignant potential (IPMN and MCN), and on the other hand, the ones with malignant potential close to 0 (PCYST and SCA). If we consider this classification, the global specificity and sensitivity for the detection of the most dangerous group were 81.8% and 97.0%, respectively, while for the least dangerous ones, they were 81.8% and 89.0%, respectively.

One of the main metrics used to evaluate the effectiveness of this method was the sensitivity or true positive rate. This is something to highlight since it is better to have a false positive than a false negative in this study due to the consequences of obtaining each one: for a false positive, a review of the detection would be needed, but for a false negative, the consequences are much worse because a PCL can exist and not be detected. If we compare the most dangerous group and the least dangerous group, meaning the one that can easily evolve to pancreatic cancer versus the one that cannot evolve to pancreatic cancer as easily, it is a remarkable fact that the sensitivity is almost 10% higher for the dangerous group, which makes the network even more efficient. Having a better true positive rate for the most dangerous group rather than for the least dangerous group is a highlight of this study.

## Discussion

### Principal Findings

In this study, we applied and validated an AGNet deep neural network to detect PCLs. The aim was to assist imaging specialists for a better diagnosis, and therefore, achieve better determining of treatment plans. First, a pancreatic CT image database with different types of cyst present was created based on the diagnosis of anatomical pathology or an imaging specialist. From this database, we established an AI system for the automatic detection of pancreatic cysts (with further classification) and then validated it in a test experiment.

In our study, the sensitivity for the detection of PCL was 93.1% (SD 0.1%), and the specificity was 81.8% (SD 0.1%), demonstrating that PCLs can be automatically detected by AI with a diagnostic performance comparable to radiologists.

This is significant because even though AI has shown excellent performance for segmentation of organs with sharp borders, in organs with vague delineation like the pancreas (eg, caused by fat interdigitations), the detection of lesions remains a difficult task for algorithms [23].

In a previous work (Abel et al [14]), an overall sensitivity of 78.8% for the detection of pancreatic cysts was obtained. The maximum sensitivity was seen in big lesions, ranging from 87.8% for cysts under 220 mm3 to 96.2% for tumors in the distal pancreas. Importantly, in this work, they analyzed the size of the lesion by volume, and in our study, we analyzed it with the diameter of the biggest slice of the lesion. Another difference between this work and ours is the deep learning architecture they used. They used an nnUNet pretrained, and we used an attention gate U-Net without pretraining.

Overall, these results demonstrate that an automated detection of PCL on CT scans is feasible.

Nevertheless, limitations to our research are still present. Although the results obtained indicate that the diagnostic accuracy is comparable to that of radiologists, it is important to bear in mind that this research intents to develop an assistive tool, not to be in any case a substitute for doctors. Moreover, this is a retrospective single-center analysis study. To further evaluate and validate the clinical applicability, next steps would include a prospective study on multicenter clinical data.

Importantly, the possibility for malignancy varies across various forms of PCLs. Therefore, precise cyst characterization is crucial for proper care. The most clinically significant distinction is separating nonmucinous cystic lesions from mucinous cystic lesions, which have malignant potential and may benefit from surgical removal. However, distinction between cyst types is difficult in a clinical setting.

Due to the lack of data for each specific subtype of PCL, this study only aimed at detecting but not classifying PCLs. Next steps would include increasing the final data set size to further assess and validate the classification performance of a deep neural network, which would have a significant effect in clinical practice.

### Limitations

PCL detection algorithm was trained and tested on data from a single hospital, which limited the available amount of data and hindered the possibility to perform an external validation.

As previously mentioned, the data in the training database were divided into 2 big groups (IPMN and MCN vs pseudocysts and SCA) due to the lack of data for each specific subtype of pancreatic cysts. For further validation, not only detection but also classification, more data are needed for the training database for each of the cyst subtypes that we are willing to differentiate.

Next steps will be to obtain images from other hardware manufacturers and improve our database. This will need to be studied thoroughly to make the images from different hospitals compatible to each other. Another approach to improve the data set is to widen the samples of each type of cyst to make it more heterogeneous.

### Conclusions

This study presents a clinical validation for automated detection of PCLs using an AGNet deep neural network. Based on the validation of an artificial deep neural network [15], results indicate that AI can be a feasible tool to help radiologist to cope with the increasing demand of cross-sectional imaging tests. The proposed method shows ability to obtain an accurate diagnosis. This artificial network, working together with specialists, proves to be a potential and effective way to tackle the early detection of pancreatic cancer.

## Abbreviations

AG: attention gate
AI: artificial intelligence
CT: computed tomography
IPMN: intraductal pseudopapillary mucinous neoplasia
HU: Hounsfield unit
MCN: mucinous cystic neoplasm
PCL: pancreatic cystic lesion
SCA: serous cystadenoma
TTA: test-time augmentation
